# Detection of human coronavirus strain HKU1 in a 2 years old girl with asthma exacerbation caused by acute pharyngitis

**DOI:** 10.1186/1743-422X-9-142

**Published:** 2012-08-03

**Authors:** Razieh Amini, Fatemeh Jahanshiri, Yasaman Amini, Zamberi Sekawi, Farid Azizi Jalilian

**Affiliations:** 1 Department of Molecular Medicine, Faculty of Medicine, Hamadan University of Medical Sciences, HUMS, Hamadan, Iran; 2 Department of Medical Microbiology, Faculty of Medicine and Health Sciences, 43400 UPM Serdang, Selangor, Malaysia; 3 Department of Infectious Disease, Masih Daneshvari hospital, Tehran, Iran; 4 Department of Medical Microbiology, Faculty of Medicine, Ilam University of Medical Sciences, 69316 IUMS, Ilam, Iran

**Keywords:** Respiratory viral infection, Asthma, Coronavirus

## Abstract

Respiratory viral infections can trigger asthma attack which may lead to sever morbidity. In this report, using molecular methods, we show the chronological association between human coronavirus - HKU1 infection and asthma exacerbation in a two years and seven months old asthmatic girl who was not under treatment and was otherwise healthy.

## Introduction

Asthma is a chronic respiratory disease characterized by hypersensitivity and inflammation of the airways with clinical symptoms of dyspnea, wheezing, coughing, reversible episodes of bronchoconstriction, shortening of breath, chronic eosinophilic inflammation, mucus hypersecretion and tightening of the chest [1,2]. The exacerbation of asthma symptoms and the level of asthma severity have shown to be related to genetic susceptibilities to exposure to various environmental triggers [3]. Inflammatory triggers of asthma include pets, dander, dust, mold, pollen, and viral respiratory infections and non-inflammatory triggers include tobacco smoke, exercise, weather changes, stress, and air pollution [4,5]. The contribution of viral infections to the occurrence of asthma exacerbations has been of broad interest for the past 20 years [6]. In this regard, it is believed that viral respiratory tract infections with respiratory syncytial virus and rhinoviruses are the major causes of acute asthma exacerbations in children [7-10]. Besides, the involvement of coronaviruses (hCoV) in asthma exacerbation has been recently revealed with the implementation of molecular methods. coronaviruses are enveloped, with a single stranded RNA, linear, non segmented and positive sense genome. These viruses are divided into 3 distinct genera (genus Alpha, Beta and Gamma coronavirus). So far, five types of human coronaviruses including HCoV-OC43 (mid-1960s), HCoV-229E (mid-1960s), SARS-CoV (2003), HCoV-NL63 (2004) and HCoV-HKU1 (2005), have been described [11]. The involvement of coronaviruses in asthma exacerbation has recently been revealed with the implementation of molecular methods such as the reverse transcriptase polymerase chain reaction (RT-PCR) [12]. Here, we report the first case of an asthma exacerbation triggered by acute pharyngitis event in a child caused by HCoV-HKU-1 infection.

## Case report

An almost three-year old girl, was admitted to the pediatric ward of Serdang Hospital (Malaysia) with a history of two days cough, runny nose and shortness of breath. Prior to the hospitalisation, she was given one dose of Salbutamol nebulizer in a private clinic for her respiratory problem. Her symptoms partially relieved. She had been diagnosed with bronchial asthma at the age of 18 months when she first presented with 2 episodes of wheezing and breathing difficulty following an upper respiratory infection which had responded well to the bronchodilator nebulization. Subsequent to the above episode, she continued to have intermittent symptoms such as nocturnal cough and occasional wheeze 2-3 times per week. However, she had not been given any prophylactic medications for asthma. The symptoms were triggered by changes in temperature, dust, common cold and cigarette smoke. Her father is a chronic smoker. There was no history of allergy but she has strong family history of asthma. There was no history of exercise-induced cough or wheeze. On admission, she was afebrile but tachypneic with respiratory rate of 50 breaths per minute with intercostal and subcostal recession. There was an audible wheeze. Her vital signs except the increased respiratory rate were within normal limits. There was no skin eczema. The patient’s throat was inflamed without tonsillar enlargement. Chest X-ray showed hyperinflation. No pneumonic changes were observed and cardiac size was normal. Generalized rhonchi on lungs auscultation could be heard.

During hospitalization she did not required oxygen. She was diagnosed with acute exacerbation of bronchial asthma due to acute pharyngitis with underlying moderate persistent asthma. She was treated with regular Salbutamol nebulisation and a five day course of oral prednisolone. She was prescribed with asthma medications including Fluticasone MDI 250 mcg to be taken twice a day and Salbutamol MDI 200 mcg to be used when needed via aerochamber device. Tamiflu syrup was also prescribed for her flu-like symptoms. An asthma education was provided to the parents. She was hospitalized for 5 days. Her blood tests revealed Hb of 12.8 g/dl, total white count of 16.2 × 10^9^/l with prodominantly neutrophil 70%, lymphocyte 21% and 7% of eosinophil. The platelet count was 390 × 10^9^/l. Her blood urea and electrolytes were within the normal limit. The level of C-Reactive Protein was 15 mg/L.

## Laboratory diagnosis

**Figure 1:**
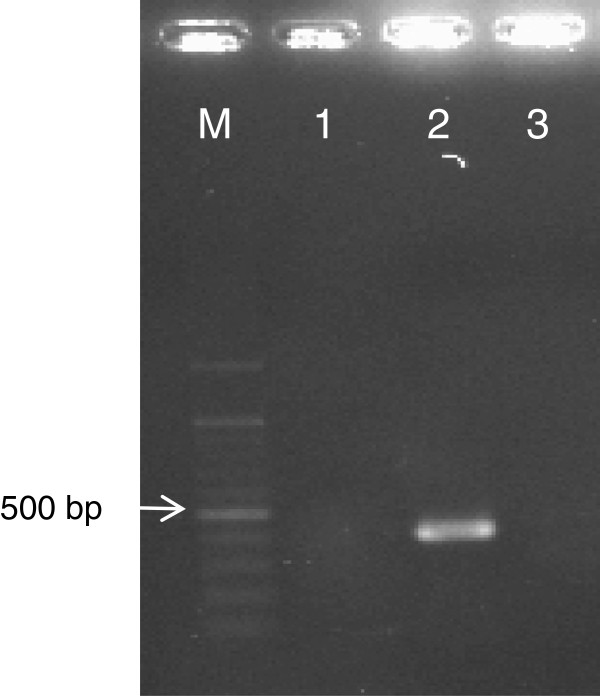
**Ethidium bromide staining of 2% agarose gel showing RT-PCR product (443 bp) of human corona virus HKU1–specific primers.** Lane M, size marker (100 bp); lane 1 Negative control RT-PCR mix; lane 2 HKU1; lane 3 OC43.

To investigate the potential causative infectious virus, the patient’s respiratory sample (NPA) was collected at the pediatrics department Serdang Hospital, Malaysia and sent to the Clinical and Molecular Virology Center (CMVC) of University Putra Malaysia. As a part of routine virological screening, DNA and RNA were extracted using viral genomic extraction kit (GeneAll, Korea) and screened for 15 respiratory viruses including influenza A and B viruses , human respiratory syncytial virus A and B viruses, para influenza 1, 2, 3 and 4 viruses, Boca virus, human metapneumovirus (hMPV), adeno virus, rhino virus, enterovirus and coronaviruses OC43/ HKU1 and NL-63/ 229E, by using a highly sensitive commercial multiplex (seeplex RV15 ACE) RT-PCR kit (Seegene, Korea) which yielded positive result for coronavirus OC43/HUK1. To differentiate between coronavirus OC43 and coronavirus HUK1 , a one-step RT-PCR amplification was carried out using RT-PCR kit (Promega, USA) with the following primer sets: HKU1( Sense 5-ACCAATCTGAGCGAAATTACCAAAC-3 and antisense 5-CGGAAACCTAGTAGGGATAGCTT-3)[11] and OC43 (Sense 5-CGATGAGGCTATTCCGACTAGGT and antisense 5-CTTGCTGAGGTTTAGTGGCAT) [13] (Figure 1).

As shown in Figure 1, RT-PCR results for HKU-1 was positive. This result was confirmed by neuclotide sequencing. To exclude bacterial infections such as mycoplasma and chlamydia pneumonia, ELISA and bacterial culture were carried out on the patient’s samples which all yielded negative results.

## Discussion

Human coronaviruses are known as the second cause of common cold [14]. Recently, HCoVs strains including 229E, OC43, NL63 and HKU1 have been shown to be associated with more severe acute lower respiratory tract infection such as pneumonia in both infants and immunocompromised patients [15,16]. These strains were also detected from asthmatic adults with ARI [17] but their relation to asthma exacerbation remains unclear [18]. It has also been shown that infection with HCoV-NL63 can preset as asthma exacerbation, croup , high fever and febrile seizures in children [19].

Another Human coronavirus strain which has been successively detected in respiratory specimen worldwide is strain HKU1. This strain was first detected by Woo et al (2005) in Hong Kong [20]. In a study on the prevalence of recently identified viruses in children with acute wheezing, HCoV-HKU1 was not detected [21]. Kuypers et al., (2007) showed that HUK1 was the most common subtype of coronaviruses found in children [22]. In a prospective longitudal cohort study of young children, NL63 and OC43 strains were shown to be associated with a burden of LRTI. Bronchiolitis, pneumonia and asthma were the most important diagnosis in these patients [23]. It has also been reported that all four strains of coronaviruses are associated with both URI and LRI in children and also OC43 and NL63 strains are responsible for the most cases of bronchiolitis, pneumonia and asthma exacerbation in these patients [24]. In this report for the first time we detected HCoV HKU1 in a child with asthma exacerbation triggered by acute pharyngitis. However, more detailed microbiological investigations especially molecular techniques are required to confirm the relationship between HCoV HKU1 infection and asthma attack. Our findings suggest the necessity of considering HCoV HKU1 as a potential cause or precursor of asthma exacerbation in children.

## Consent

Written informed consent was obtained from the patient’s parents for publication of this Case Report. A copy of written consent is available for review by the Editor- in- Chief of this journal.

## Competing interests

The authors declare that they have no competing interests.

## Authors’ contribution

R.A carried out the molecular studies and participated in the sequence alignment and drafted manuscript, F.J performed the ELISA and participated in drafted the manuscript, Y.A carried out the clinical diagnosis and participated in the design of the study, Z.S performed the bacterial culture and participated in the clinical diagnosis, F.A.J conceived of the study, and participated in its design and coordination and helped to draft the manuscript. All authors read and approved the final manuscript.
